# Eco-Friendly Synthesis of Silver–Cellulose Nanocomposite Adsorbent from Agricultural Residues for Binary Dye System Remediation

**DOI:** 10.3390/polym17182555

**Published:** 2025-09-22

**Authors:** Doaa S. Al-Raimi, Reem M. Alghanmi, Ghalia S. Aljeddani, Ragaa A. Hamouda

**Affiliations:** 1 Department of Chemistry, College of Science, University of Jeddah, P.O. Box 80327, Jeddah 21589, Saudi Arabia; rmalghanmi@uj.edu.sa; 2 Department of Environmental Science, College of Science, University of Jeddah, P.O. Box 80327, Jeddah 21589, Saudi Arabia; gsalgdanee@uj.edu.sa; 3 Department of Applied Radiologic Technology, College of Applied Medical Sciences, University of Jeddah, P.O. Box 80327, Jeddah 23218, Saudi Arabia; 4 Microbial Biotechnology Department, Genetic Engineering and Biotechnology Research Institute, University of Sadat City, Sadat City 32897, Egypt

**Keywords:** peanut shells, micro-cellulose, silver nanoparticles, nanocomposite, methylene blue, safranin O, adsorption, binary-system

## Abstract

This work reports a one-step, green synthesis of silver-micro cellulose nanocomposite (Ag@Ce NCs) using *Azadirachta indica* A. Juss leaf extract to load micro-cellulose isolated from peanut shells with silver nanoparticles, followed by comprehensive physicochemical characterization (FTIR, TEM, EDX-SEM, zeta potential, and XRD). The composite has pH_PZC_ ≈ 5.0 and was tested for simultaneous removal of methylene blue (MB) and safranin O (SO) under batch conditions across various pH levels, doses, contact times, initial concentrations, ionic strengths, and temperatures. The high removal efficiencies observed at pH 10 for MB and 6.0 for SO. The adsorption reached the maximum at 45 min before partially declining, indicating reversible binding on saturated surfaces. Isotherm study favored the Langmuir model, with similar affinities (*K_L_* ≈ 0.106, and 0.110 L/mg) and monolayer capacities of 17.99 mg/g for MB and 14.90 mg/g for SO, suggesting non-selective competition on uniform sites. Kinetic data fitted the pseudo-second-order model, while thermodynamic analysis indicated mainly exothermic and physisorption interactions. Higher ionic strength reduced removal efficiency (at 1.0 M NaCl, %*RE* ≈ 33–48%), highlighting salt sensitivity typical of electrostatic attraction. The adsorbent maintained about 90% of its initial performance after five adsorption–desorption cycles in 0.1 M H_2_SO_4_, indicating excellent reusability. Overall, Ag@Ce NCs provide an inexpensive, eco-friendly, and reuseable platform for treating binary mixtures of cationic dyes.

## 1. Introduction

Textile wastewater contains large amounts of dyes, azo dyes, and dyestuff, which contribute to environmental contamination. Discharging effluents containing various dyes into water is harmful due to their severe toxic effects on humans and the aquatic ecosystem. Several printing, food, leather, paper, and textile facilities generated more than 100,000 tons of azo dye and dye-related products worldwide. These dyes were released into the environment and caused numerous health issues [1]. Covenantal treatments, such as coagulation, filtration, and precipitation, are used on wastewater containing dyes through physicochemical techniques [2]. While these physicochemical procedures are quick and effective, they often fail due to their high costs and complex sludge formation [3].

Cellulose nanocrystals (CNCs) and cellulose nanofibrils (CNFs) are two general types of cellulose nanomaterials (CNs) that can be recovered from various resources such as plants, animals, or mineral plants. Cellulose nanowhiskers (CNWs) or nanocrystalline cellulose (NCC) may also be referred to as cellulose nanocrystals [4]. Cellulose and nanocellulose can be synthesized from agro-waste due to the significant amount of agro-waste produced globally, which, if not managed properly, can contaminate the environment and pose health risks. Cellulose nanocomposites and nanocellulose exhibit unique properties, including being renewable materials, highly available, mechanically strong, featuring high aspect ratios, dimensional stability, biocompatibility, and biodegradability. Various nanocrystalline cellulose (NCC) types have been employed for dye removal, including those made from wastepaper, agroforestry, and wood [5]. Since nanocellulose has been utilized as an adsorbent in many wastewater treatment processes, including the removal of dyes and heavy metals, it is essential to explore new methods for dye extraction from wastewater [6]. Farming activities that generate agro-waste include horticulture, seed production, dairy farming, animal breeding, grazing, nursery plots, market gardens, and forestry or woodland production [7]. There are three categories of agricultural waste: liquid, solid, and slurry [8]. Agro-waste constitutes about 30% of the agricultural products generated globally. Agricultural leftovers can include animal and plant byproducts (such as manure), crop remnants, byproducts from other agricultural processes (like pruning, harvesting, growing, and fertilizing), and pesticides used in the production of these goods. Many studies have examined the negative impacts of agricultural waste on the environment, such as reducing global nutrition and damaging ecosystems through food waste [9]. Currently, the poor handling of agricultural waste contributes to numerous environmental problems, primarily due to field burning. The burning of agro-waste has a substantial impact on human health, environmental quality, and climate change [10]. Underutilized agro-waste is a crucial source of lignocellulosic materials, including common examples such as millet, rice, wheat, corn straw, cocoa husk, corncobs, and fiber [11]. Peanut shells (PNSs) are a significant agricultural waste produced in large quantities each year [12]. Peanut shells can be considered a candidate for cellulose extraction, as they contain approximately 35.7% cellulose and 18.7% hemicellulose of the total dry weight [13]. Two methods have been employed to obtain cellulose nanocrystals from peanut shells: alkali hydrolysis and sulfuric acid hydrolysis, followed by bleaching with hydrogen peroxide or sodium chlorite [14].

The concept of nanocomposites integrates various materials, including at least one material in the nanoscale range [15]. They are widely utilized to combine metal nanoparticles (Ag, Cu, Au, Cd, etc.), either alone or with polymers, to ensure their homogeneous integration in producing nanocomposites [16]. High-performance materials with unique qualities are referred to as nanocomposites [17]. Due to the unique properties of nanocellulose and cellulose nanocomposites, they have attracted wide interest for environmental remediation [18]. For instance, nanocrystalline cellulose (NCC) has been reported to remove negatively charged dyes from aqueous solutions [19], while silver–cellulose nanocomposites have shown efficiency in adsorbing malachite green (MG) dye through the activity of silver nanoparticles [20]. Building on this foundation, our previous work demonstrated the preparation of a silver–cellulose nanocomposite from peanut-shell-derived micro-cellulose, which was successfully applied for the cost-effective removal of crystal violet dye [21]. These examples highlight the promising role of cellulose-based nanocomposites in dye removal and provide the basis for the present study.

Given that most industrial wastewater contains numerous contaminants, it is essential to investigate the impact of multi-component systems on adsorption capacities. Several studies have explored the simultaneous removal of multiple contaminants from aqueous solutions to evaluate the competitiveness of adsorbates [22]. In contrast to our previous work [21], this study prepared the silver-cellulose-nanocomposite (Ag@Ce NCs). The present study differs from earlier research in several ways: (i) the composite is prepared in one step instead of two; (ii) it examines the adsorption performance of two different dyes simultaneously, methylene blue (MB) and safranine O (SO) (binary dye system). We investigated various parameters that influence adsorption performance. The experimental results were predicted using kinetic and isothermal models. The adsorption mechanism of the dyes was explained based on data from surface chemical characterization and analytical devices, including FTIR, TEM, EDX-SEM, XRD, and Zeta. This study is crucial for advancing practical and eco-friendly adsorbents for the mixture of organic contaminants

## 2. Materials and Methods

### 2.1. Materials

All the chemical reagents used were obtained from Sigma-Aldrich (St. Louis, MO, USA). Glacial acetic acid (CH_3_COOH), ethanol (EtOH, 96%), hydrochloric acid (HCl; 0.1 M), nitric acid (HNO_3_; 69%), silver nitrate (AgNO_3_; 1.0 mM), sodium hydroxide (NaOH; 0.5 M and 0.1 M), and sodium hypochlorite solution (NaOCl; 10%) were used as received without any purification. Methylene blue and safranin O dyes served as adsorbates, and their main characteristics are illustrated in Table 1. All reagents and dye solutions were prepared in double-distilled water (DDW). Peanut shells (PNS), used as the primary source of cellulose in this study, were procured from a nearby market in Jeddah, Saudi Arabia. PNS was washed several times with DDW to remove impurities and then dried in an oven for 24 h at 60 °C. The dried PNS was ground into powder and then sieved to create uniform particles. Fresh *A. indica* leaves were collected from neem trees in Jeddah. The aqueous extract of the leaves was prepared by boiling 10 g of clean, dried, and chopped *A. indica* leaves in 75 mL of DDW for 10 min, followed by filtration after cooling. The fresh aqueous extract was stored in a dark bottle at 4 °C for over three days.

### 2.2. Isolation of Micro-Cellulose from PN Shells

Micro-cellulose was extracted from PNS using the modified methods reported in the literature [21,23]. Approximately 25 g of PNS powder was mixed with 750 mL of 0.5 M NaOH and stirred for 24 h at 90 °C. The mixture was then filtered. The dark slurry was discarded, and the remaining residue was rinsed several times with DDW before being dried. The treated PNS was refluxed with a 20% (*v*/*v*) solution of HNO_3_ and EtOH for 7 h until the reaction color changed from brown to yellow. The yellow product was filtered and rinsed with cold DDW until the filtrate reached neutrality. The yellow extracted micro-cellulose was decolorized using NaOCl and drops of acetic acid. Finally, the micro-cellulose was dried at 60 °C for 24 h in an oven. The process involves the selective removal of non-cellulosic components. Alkaline pretreatment with NaOH hydrolyzes ester bonds and removes hemicellulose, while the HNO_3_/EtOH reflux step promotes further delignification through oxidative cleavage of lignin structures. The subsequent bleaching with NaOCl and acetic acid decolorizes the material by oxidizing residual lignin chromophores. These treatments together expose the cellulose microfibrils, resulting in purified micro-cellulose with reduced lignin and hemicellulose content [13,14,23].

### 2.3. Synthesis of Ag@Ce NCs

The silver-cellulose nanocomposite (Ag@Ce NCs) was synthesized in one step by mixing 5.0 mL of *A. Indica* extract with 2.0 g of isolated micro-cellulose, and adding 95 mL of 1.0 mM AgNO_3_ dropwise from a burette to the mixture while stirring for one hour at 80 °C. The color of the mixture changed to dark brown instead of light green, indicating the formation of Ag-NPs. The mixture was stirred continuously for 24 h. The silver–cellulose nanocomposite was separated from the solution by centrifuging at 10,000 rpm. The nanocomposite (Ag@Ce NCs) was dried in an oven at 60 °C until it reached a constant weight.

### 2.4. Characterization of Ag@Ce NCs

The FTIR spectra of raw PNS powder, extracted micro-cellulose, Ag@Ce NCs, and Ag@Ce NCs loaded with adsorbed dyes were recorded as KBr disks using a Frontier Fourier transform infrared spectrophotometer (Perkin-Elmer, Waltham, MA, USA) to identify the functional groups present in these analytes. A field emission scanning electron microscope (SEM) integrated with energy-dispersive spectroscopy (EDX) (JEOL JSM-6510/v, Tokyo, Japan) was utilized to determine the elemental composition of the Ag@Ce NCs. Transmission electron microscopy (TEM) was employed to examine the particle size and morphology of the nanocomposite. The stability of the synthesized Ag@Ce NCs was analyzed using zeta potential assessment (Malvern Zeta size Nano-Zs90, Westborough, PA, USA). The X-ray diffraction pattern was recorded using an X-ray diffractometer (PAN Analytical X-Pert PRO, Spectral plc, Almelo, The Netherlands) to investigate the crystallinity of Ag-cell-NCs.

### 2.5. Adsorption and Desorption Studies

**Figure 1:**
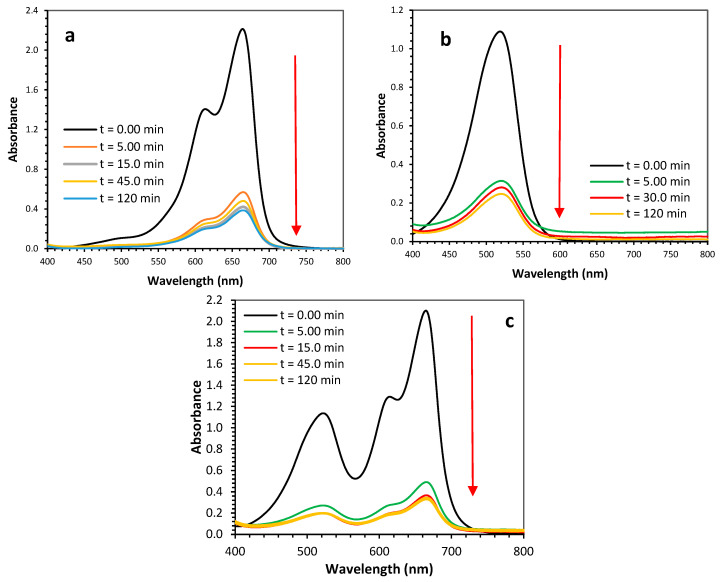
UV-Vis absorption spectra for aqueous solutions of MB dye (**a**), SO dye (**b**), and binary-adsorption system of MB and SO (**c**), along with Ag@Ce NCs after various contact times (*C*_0_ = 15 mg/L, *m* = 20 mg, *V* = 10 mL, pH = 6.8, AS = 200 rpm, *T* = 293 K).

The adsorption and desorption studies were conducted using a batch experimental process. For the adsorption studies, 10 mL of dye solution (MB or SO or a mixture of MB and SO) at the desired initial concentration (5 to 30 mg/L) and pH = 6.8 was equilibrated in 100 mL conical flasks with 0.02 g of Ag@Ce NCs in a shaker at 200 rpm under room temperature conditions. The effects of various parameters on the adsorption process were investigated, including the pH of the initial solution, contact time (in minutes), adsorbent dose (g/L), initial dye concentration (*C*_0_, mg/L), and temperature (*T*). At equilibrium, the dye solution was separated from the adsorbent using a centrifuge at 4000 rpm. The residual concentration was determined using a UV-Vis spectrophotometer (Shimadzu 1800 UV, Japan) at the wavelength corresponding to the maximum absorbance of MB (λ_max_ = 664 nm) and SO (λ_max_ = 522 nm), as shown in Figure 1. The removal efficiency (%*R*) and the equilibrium adsorption capacity (*q_e_*, mg/g) were calculated by applying Equations (1) and (2).

(1) $$\%RE=\frac{\left(C_{0}-C_{e}\right)}{C_{0}}\times 100$$

(2) $$q_{e}=\frac{(C_{0}-C_{e})\times V}{m}$$

where *C*_0_ and *C*_e_ represent the dye concentrations in the solution (mol/L) before and after adsorption, respectively; *V* is the volume of the solution (L); and m is the dosage of the Ag@Ce NCs (g/L). To ensure the highest level of precision and minimize the possibility of errors, all batch adsorption experiments were conducted in triplicate, and the standard deviation was calculated. Some of the UV-Vis absorption spectra measured during the study of the different parameters (adsorbent dosage, contact time, initial dye concentration, ionic strength, and temperature) are shown in the Supplementary Materials (Figures S1–S5).

For reusability experiments, each adsorption–desorption cycle was carried out under identical conditions to ensure comparability. In each run, 50 mg of Ag@Ce NCs were added to 10 mL of the binary solution of MB/SO (initial concentration of 15 mg/L of both dyes) at natural pH (≈6.8), then the mixture was stirred at 200 rpm for 24 h at room temperature (293 K). Afterward, the Ag@Ce NCs were separated using centrifugation, and the concentration of the dyes in the remaining solution was measured. The resulting Ag@Ce NCs were regenerated using 50 mL of 0.1 M H_2_SO_4_, then thoroughly rinsed with DDW, and dried in an oven at 60 °C before reuse. The regenerated adsorbents were reused for the removal of MB and SO, with the process repeated five times to assess their recyclability. The removal efficiency (*RE*, %) and desorption efficiency (*DE*, %) were calculated for each cycle.

(3) $$\%DE=\frac{R}{D}\times 100$$

where *R* and *D* are dye-recovered (desorbed) and dye-adsorbed, respectively.

### 2.6. Adsorption Kinetics

To investigate the kinetics of the adsorption process for MB and SO dyes on Ag@Ce NCs, the pseudo-first-order (PFO) [24], and pseudo-second-order (PSO) models [25] were applied using Equations (4) and (5), respectively.

(4) $$\ln\left(q_{e}-q_{t}\right)=lnq_{t}-k_{1}t$$

(5) $$\frac{t}{q_{t}}=\frac{1}{k_{2}\cdot q_{e}^{2}}+\frac{1}{q_{e}}t$$

where *q_e_* and *q_t_* (mg/g) are the amount of adsorbates at equilibrium and at any time *t*, respectively; *k*_1_ and *k*_2_ are the rate constants of PFO and PSO, respectively. The rate constants $k_{1}$ , and $k_{2}$ can be obtained by a linear plot of *ln* ( $q_{e.exp}-q_{t}$ ) and *t*/ $q_{t}$ against time, respectively.

### 2.7. Adsorption Isotherms

The adsorption isotherm provides important information about the behavior of the adsorbent surface. It is used to understand how adsorbate molecules are distributed between the liquid and solid phases at equilibrium, and to find a suitable model for design purposes [26]. Different isotherm models, namely Langmuir, Freundlich, and Temkin isotherm models, were employed to evaluate the adsorption process of MB and SO dyes on Ag@CE NCs [27]. By analyzing the results of the linear regression correlation coefficient (R^2^), the best-fit model was chosen. The mathematical equations for the isothermal models applied are presented in Table 2.

### 2.8. Thermodynamic Parameters

Important information about the energy changes during adsorption can be obtained by determining the thermodynamic parameters. The Van’t Hoff equation is used to calculate the conventional thermodynamic parameters [31], including free energy (Δ*G°*) [32], enthalpy (ΔH), and entropy (ΔS), based on how temperature affects the adsorption process. In this study, the distribution coefficient, *K_d_*, was used to estimate these parameters, as reported in several previous works [21,26,33,34]. This method offers an approximate assessment of spontaneity and energy change, even though it does not produce a dimensionless standard equilibrium constant.

(10) $$∆G{}^{\circ}=-RT\ln K_{d}$$

(11) $$K_{d}=\frac{q_{e}}{C_{e}}$$

(12) $$lnK_{d}=\frac{{∆S}^{{}^{\circ}}}{R}-\frac{{∆H}^{{}^{\circ}}}{RT}$$

Here, *R* stands for the universal gas constant (8.314 J/K mol), and *T* is the temperature on the Kelvin scale, while *K_d_* is the distribution coefficient.

## 3. Results and Discussion

### 3.1. Characterization of Ag@Ce NCs

#### 3.1.1. Point of Zero Charge (pH_pzc_)

**Figure 2:**
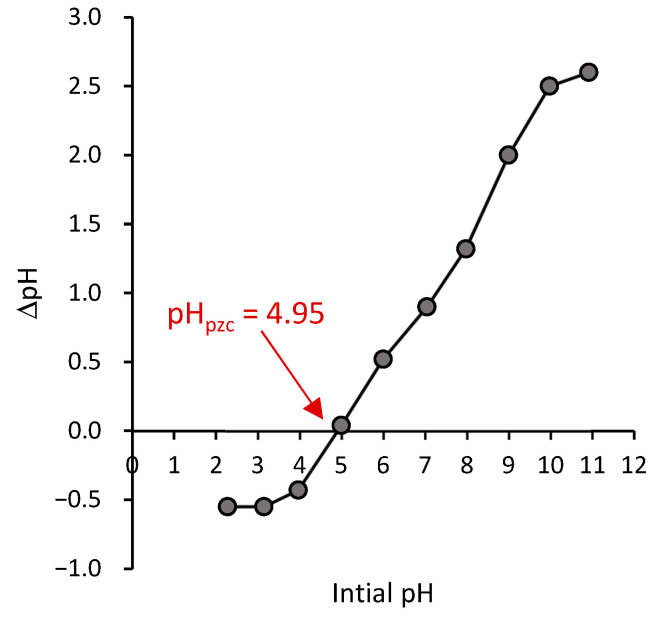
Point zero charge of the Ag@Ce NCs.

The surface charge on the adsorbent could be examined by determining the point of zero charge using the method described in the literature [35]. Figure 2 shows the experimental result of the pH_PZC_ plot, indicating that the point of the zero charge for the Ag@Ce NCs is approximately 5, at which the ΔpH equals zero, where the plot intersects with the x-axis. This pH_PZC_ is very close to the previously published value for cellulose loaded with silver nanocomposites [21]. The point of zero charge (pH_pzc_) is defined as the point at which the total positive and negative charges on the surfaces of the used adsorbent are equal [36]. The value of pH_pzc_ aids in understanding the interactions on the surface of materials, particularly for charged adsorbents, where electrostatic interactions dominate the adsorption mechanism.

#### 3.1.2. FTIR Spectra

**Figure 3:**
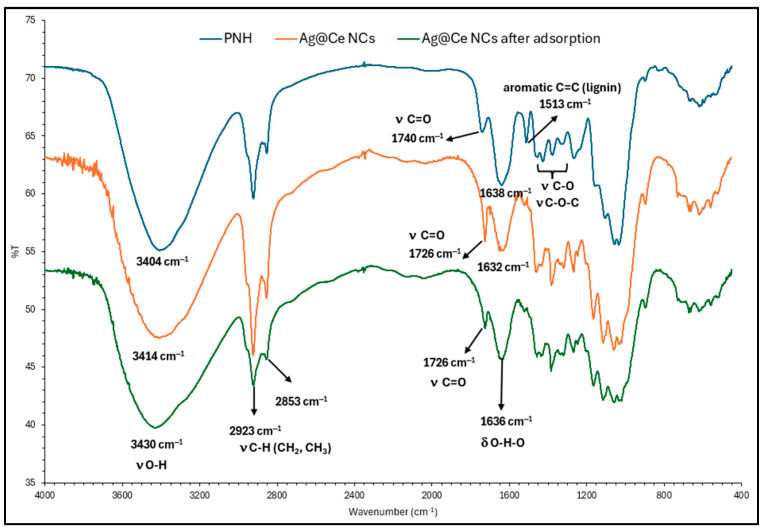
FTIR spectra of raw PNH, Ag@Ce NCs, and Ag@Ce NCs after adsorption.

Figure 3 shows the FTIR spectra of the raw PNS and the Ag@Ce NCs before and after adsorption of MB and SO dyes. The raw PNS spectrum shows the characteristic lignocellulosic peaks: a broad O–H stretch at 3404 cm^−1^ from hydrogen-bonded hydroxyl groups in cellulose/hemicellulose, aliphatic C−H stretches at ~2923 and ~2853 cm^−1^, a carbonyl peak at ~1740 cm^−1^ attributed to unconjugated C=O groups in hemicellulose acetyl/ester moieties, an H−O–H bending vibration peak of adsorbed water at ~1638 cm^−1^, and a lignin aromatic skeletal vibrations at ~1513 cm^−1^. The carbohydrate backbone (C–O and glycosidic C–O–C) is confirmed by the peaks at ~1457, 1378, and 1324 cm^−1^; a weak aromatic out-of-plane signal appears at ~618 cm^−1^. After cellulose isolation and Ag loading (Ag@Ce NCs), the O–H peak blue-shifts from ~3404 to 3414 cm^−1^, indicating a slight weakening and rearrangement of the hydrogen-bond network due to the surface polarization and weak Ag-O interactions at hydroxyl/ether sites. Simultaneously, the hemicellulose carbonyl at ~1740 cm^−1^ decreases and shifts to ~1726 cm^−1^, and the lignin marker at ~1513 cm^−1^ disappears, indicating effective delignification and partial hemicellulose removal. Meanwhile, carbohydrate peaks at 1267, 1165, and 1115 cm^−1^ intensify and shift relative to PNS, reflecting perturbation of C–O and C–O–C linkages by Ag incorporation. A peak at 897 cm^−1^, assigned to the amorphous cellulose region and β-glycosidic linkages, is also evident. Following the adsorption of MB and SO dyes onto Ag@Ce NCs, only minor changes in the peak positions are observed (Figure 3), indicating minimal alteration in the chemical structure of Ag@Ce NCs before and after dye adsorption. Notably, a shift in the O–H stretch to ~3430 cm^−1^, without the appearance of new peaks or loss of the cellulose backbone, indicates that dye adsorption proceeds primarily through nanocovalent interactions (hydrogen bonding and electrostatic attractions at surface hydroxyl/ether groups) while the cellulose framework remains intact. Previous studies showed essentially unchanged FTIR spectra for Ag-loaded cellulose after the adsorption of crystal violet dye (CV), and for cellulosic olive-stone biomass following the adsorption of MB dye.

#### 3.1.3. TEM Analysis

**Figure 4:**
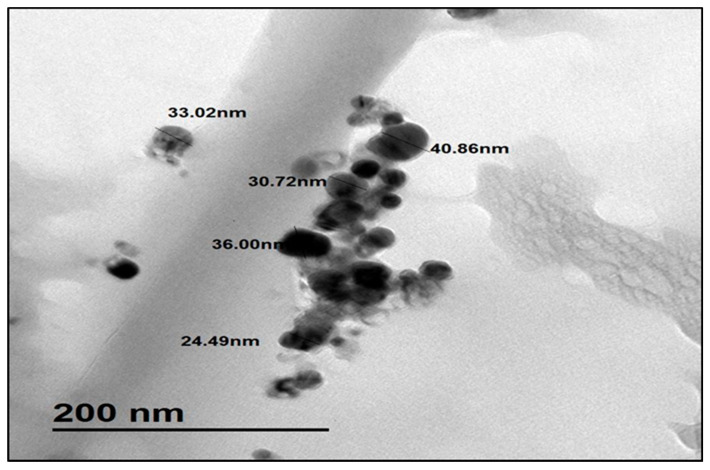

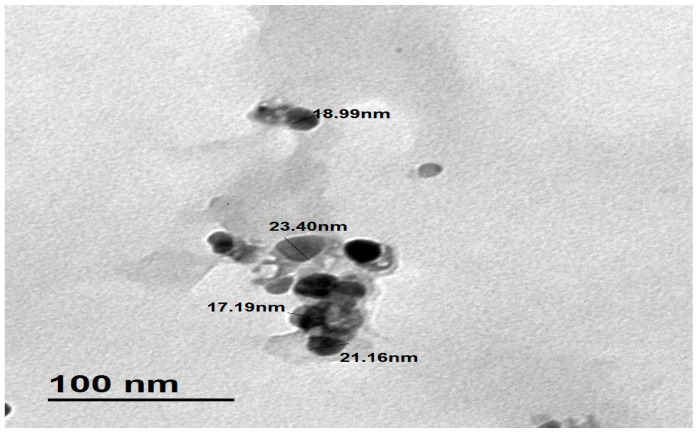
TEM image of the Ag@Ce NCs.

The TEM image of Ag@Ce NCs is demonstrated in Figure 4, displaying mostly spherical particles with sizes ranging from 24.49 to 40.86 nm, corresponding to the silver nanoparticles, while the arrows indicate implanted nanocellulose. This particle size range and spherical morphology are consistent with previous studies on plant-derived nanocellulose loaded with Ag nanoparticles, which commonly report well-dispersed spherical Ag NPs in the range of 18–45 nm [21,37,38], though smaller than Ag NPs grown longer on cellulose fibers (46–75 nm) [39]. The spherical shape and presumably uniform distribution suggest effective stabilization by the cellulose matrix, similar to other nanocellulose-Ag systems.

#### 3.1.4. EDX-SEM Analysis

**Figure 5:**
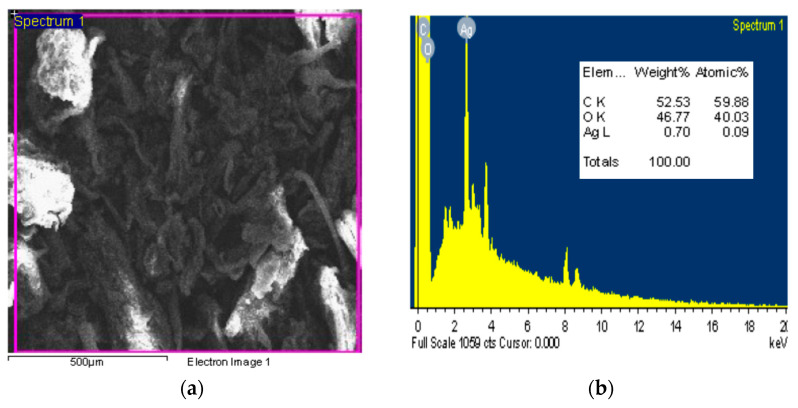
SEM image (**a**), and EDX (**b**) analysis of the Ag@Ce NCs.

The SEM image and EDX measurements of the Ag@Ce NCs are shown in Figure 5. The SEM image shows that the nanocellulose has a uniform nanofibrous morphology with a smooth surface, free from cracks or voids. The Ag-NPs appear randomly distributed along the cellulose network, consistent with previous observations [40]. These nanofibers have consistent diameters with a smooth surface [41]. The EDX analysis indicates that the Ag@Ce NCs contain carbon, oxygen, and silver, which represent weight percentages of 52.53%, 46.77%, and 0.70%, respectively. The high content of carbon and oxygen in the Ag-cellulose nanocomposite reflects the natural composition of cellulose, where carbon forms the main backbone and oxygen exists in hydroxyl and ether groups. These results align with previous elemental analyses, such as EDX, of Ag-cellulose-based materials, which consistently show dominant carbon and oxygen, with silver typically present in trace amounts not exceeding a few weight percent [21,42,43,44].

#### 3.1.5. Zeta Potential Analysis

**Figure 6:**
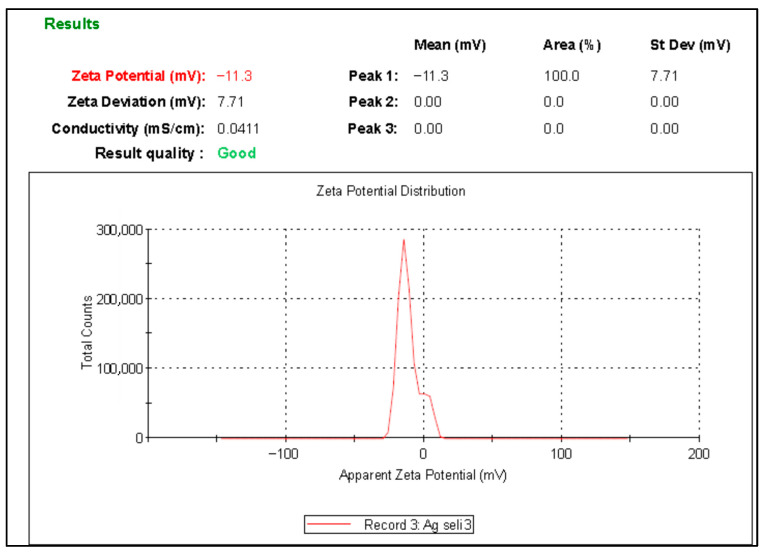
Zeta potential analysis of the Ag@Ce NCs.

The zeta potential analysis of Ag@Ce NCs is shown in Figure 6. The results indicate that Ag@Ce NCs have a negative surface charge of −11.3 mV, when measured at pH 7. This negative charge provides important insights into their surface chemistry and colloidal stability. A negative zeta potential means that the particles are surrounded by an excess of negative charges on their surface. In aqueous systems, this typically results from the adsorption of hydroxide ions or the dissociation of acidic surface groups [45]. Zeta potential values closer to (e.g., −11.3 mV) suggest moderate to low stability. Such particles may tend to aggregate over time because weaker repulsive forces are unable to fully prevent particle interactions [46]. These findings confirm that the Ag-cellulose particles are generally negatively charged, which is essential for understanding their aggregation behavior, colloidal stability, and interactions in various applications [47].

#### 3.1.6. XRD Pattern Analysis

**Figure 7:**
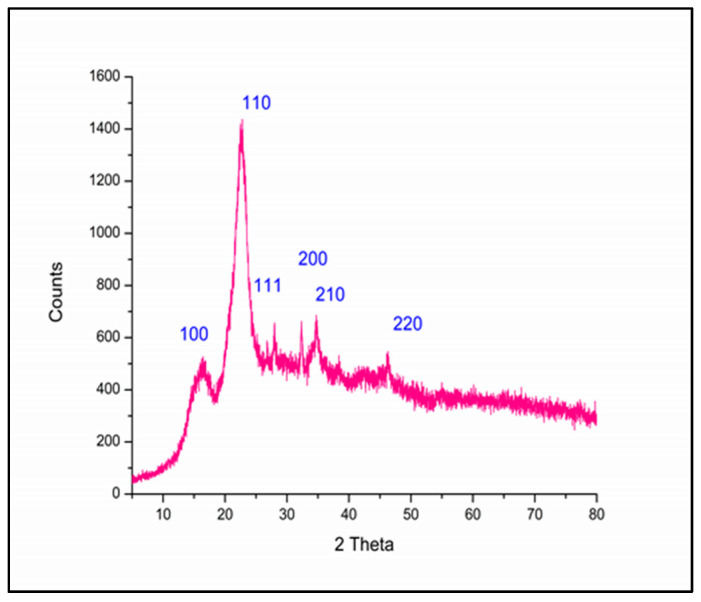
XRD pattern of the Ag@Ce NCs.

Figure 7 illustrates the crystalline structure of Ag@Ce NCs, as examined through XRD measurements. The peaks appear at 16.353°, 22.608°, 27.944°, 32.282°, 34.766°, and 46.252°, which can be assigned to the Miller indices (hkl) 100, 110, 111, 200, 210, and 200, respectively [21]. These peaks correspond to diffraction from specific crystalline planes of metallic silver and confirm the successful incorporation of Ag-NPs within the composite matrix. Some variations in peak positions and intensities may occur based on the synthesis conditions and size of the Ag-NPs [48]. The results of the diffraction patterns demonstrate features typical of Ag-cellulose nanocomposites, including crystalline peaks from metallic Ag-NPs (primarily (111), (200), (220)) embedded in the semi-crystalline cellulose network (broad peaks and lower angles). Collectively, these patterns confirm the successful formation of a nanocomposite with both components contributing to its overall structure [43,49].

#### 3.1.7. The Mechanism of Ag@Ce NCs Formation

Ag@Ce NCs are formed through a cooperative process involving coordination, reduction, and stabilization. Silver ions initially attach to micro-cellulose functional groups, such as hydroxyls and oxidized carboxylates, forming nucleation complexes [50,51,52]. In the presence of *A. indica* leaf extract, phytochemicals like diterpenoids, phenolics, and flavonoids are introduced. These compounds act as capping agents and electron donors, converting Ag⁺ to metallic Ag (Ag^0^). They adhere to the nanoparticle surface, maintaining colloidal stability and preventing uncontrolled aggregation [53]. The overall process involves in situ Ag⁺ binding, local electron transfer, and the growth of surface-anchored nuclei into uniformly dispersed nanoparticles embedded within the cellulose framework. Beyond its role in anchoring and stabilization, cellulose provides additional advantages for dye removal: its abundant surface hydroxyl and carboxyl groups enhance dye adsorption and concentrate dye molecules close to Ag catalytic sites, thereby accelerating removal efficiency. Moreover, micro-cellulose prevents nanoparticle agglomeration and leaching, ensuring reusability, while offering sustainability, low cost, and biocompatibility. The collaboration between micro-cellulose adsorption and Ag NP catalytic and plasmonic activity results in a composite that is more effective, stable, and environmentally friendly than either component alone [51,54,55].

### 3.2. Adsorption Study

#### 3.2.1. Effect of pH

**Figure 8:**
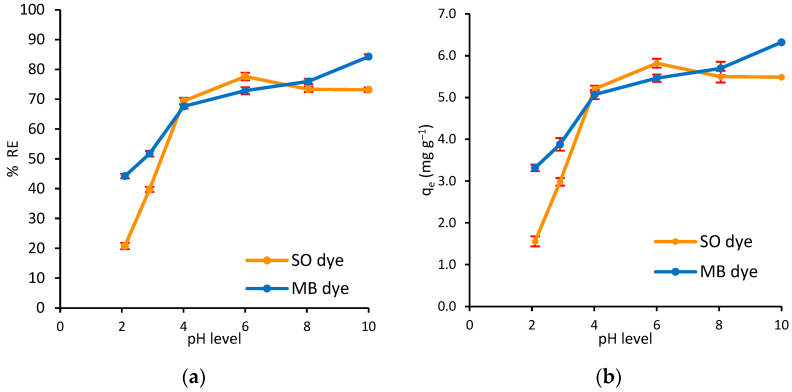
Effect of pH level on the (**a**) adsorption capacity, (**b**) simultaneous removal efficiency of MB and SO dyes onto Ag@Ce NCs (*C*_0_ = 15 mg/L, *m* = 20 mg, *V* = 10 mL, *t* = 45 min, AS = 200 rpm, *T* = 293 ± 1 K).

The adsorption process is highly dependent on the initial pH of the solution. The pH affects the amount of dyes adsorbed onto the adsorbent because it controls the ionization of the dyes and the charge of the adsorbent surface [56]. The adsorption of MB and SO onto Ag@Ce NCs in the binary system was investigated over a pH range of 2 to 10. The initial pH value of the solutions was adjusted by adding 0.1 M HCl or 0.1 M NaOH as needed. It is worth mentioning that changing the pH of the dye solution does not affect its color. Figure 8a,b show the effect of varying pH on the percentage of dye removal (%*RE*) and the adsorption capacity (*q_e_*), respectively. The results in Figure 8 indicate that the adsorption process for both dyes in the binary adsorption system gradually increased with rising pH. For MB dye, the maximum removal was achieved at a pH level of 10 (%*RE* = 84.3%, and *q_e_* = 6.32 mg/g). The maximum removal of SO dye occurred at a moderate pH level (%*RE* = 77.6%, and *q_e_* = 5.82 mg/g at pH 6), after which it remained relatively constant as the pH increased until pH 10.

Based on the experimental value of pH_pzc_, the charge of the Ag@Ce nanocomposite is positive at pH < 5, and it is negative at pH > 5. This indicates that increasing the pH level of the solution enhances the efficiency of the adsorption process as the negatively charged composite surface attracts the dye cations. Consequently, the removal of both dyes from the solution increases. On the other hand, the addition of HCl to adjust the acidic medium causes protonation of the Ag@Ce NCs surface, leading to a repulsive force that hinders the effective adsorption of cationic molecules of MB and SO dyes. Conversely, in basic medium, adding NaOH deprotonates the adsorbent surface, which enhances the electrostatic attraction between the negatively charged surface and the cationic dyes, thereby facilitating more effective binding of the dyes. Consequently, the simultaneous removal of MB and SO dyes from the solution significantly increases under basic conditions. The lower adsorption notice in acidic conditions can be attributed to competition between cationic dye molecules and hydrogen ions for available active sites on the adsorbent. This indicates that electrostatic interactions between the dye molecules and the surface of Ag@Ce NCs play a crucial role in the adsorption process [57].

Finally, the difference in the optimal pH for each dye in the binary system can be explained by the ionization constant of each dye (pKa of MB = 3.8 [58], and SO = 6.4 [59]). When MB and SO are present together, they compete for the same negatively charged sites. Under alkaline conditions (pH ≈ 10), MB retains its full positive charge and binds more effectively due to increased surface negativity [60], while SO becomes less charged and less competitive. At a pH of about 6, both dyes are fully protonated, which increases the competition [59]. MB generally dominates in alkaline media because of its stable charge, whereas SO performs better in mildly acidic conditions. This competition often reduces the adsorption capacity of each dye compared to when they are alone. Since MB and SO have different optimal pH values, conducting the next experiments at two separate pH levels would not accurately represent their simultaneous removal. Therefore, the natural pH of the mixed dye solution (≈6.8) was chosen as a compromise, and all subsequent parameter studies were performed at this pH.

#### 3.2.2. Effect of Adsorbent Dosage

**Figure 9:**
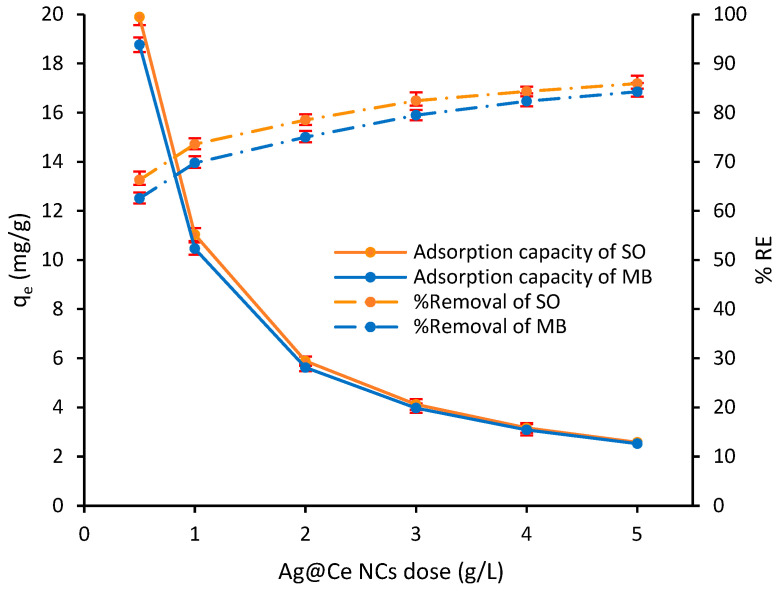
Effect of Ag@Ce NCs dosage on the adsorption capacity, simultaneous removal efficiency of MB and SO dyes (*C*_0_ = 15 mg/L, *V* = 10 mL, pH = 6.8, *t* = 45 min, AS = 200 rpm, *T* = 293 ± 1 K).

The adsorbent is a critical parameter that influences the efficiency of the adsorption process, as it defines the ratio between the amount of adsorbent and the quantity of adsorbate in the system. In this study, the effect of Ag@Ce NCs dosage was assessed across a range of 1.0 to 5.0 mg/g, with an initial concentration of 15 mg/L for both MB and SO dyes in the binary solution. As shown in Figure 9, increasing the dosage to 5.0 mg/g resulted in removal efficiencies of 84.3% for MB and 85.9% for SO, indicating a significant improvement in performance with a larger quantity of adsorbent. This improvement can be attributed to the increased availability of active surface areas and adsorption sites, facilitating more effective dye uptake under constant contaminant concentrations. However, the increase in adsorbent dosage led to a decrease in equilibrium capacity (*q_e_*). Specifically, *q_e_* decreased from 18.8 to 2.53 mg/g for MB and from 19.9 to 2.53 mg/g for SO as the amount of Ag@Ce NCs increased (Figure 9). This phenomenon is consistent with Kroeker’s rule, which states that increasing the mass of the adsorbent at a fixed initial adsorbate concentration results in a lower specific adsorption volume [61]. Additionally, the reduced adsorption capacity may be due to adsorbent aggregation or overlapping of active sites at higher concentrations, which limits the accessible surface area. Moreover, in more concentrated suspensions, some adsorption sites or surface functional groups may remain unsaturated, further contributing to a decline in specific adsorption [61,62,63].

An interesting observation from Figure 9 is that as the Ag@Ce NCs dose increases while maintaining the dye concentration constant, the *q_e_* for MB and SO in the binary solution becomes almost identical. This is due to the increasing number of active sites relative to the available dye molecules, leading to an unsaturated adsorbent surface and similar dye uptake per gram. Literature confirms that in competitive systems with excess adsorbent, structurally similar dyes often show similar adsorption behavior [60,64]. Additionally, high adsorbent doses are known to reduce the per-gram capacity because of the availability of unsaturated sites and potential adsorbent aggregation, which supports the near-equal *q_e_* values.

#### 3.2.3. Effect of Contact Time

**Figure 10:**
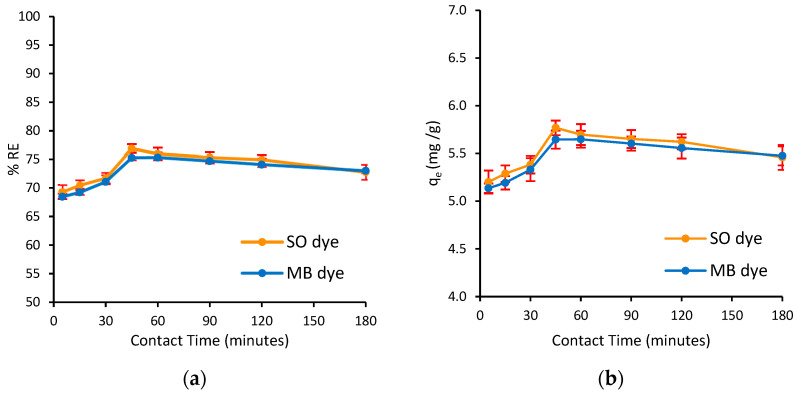
Effect of contact time on the (**a**) adsorption capacity and (**b**) simultaneous removal efficiency of MB and SO dyes onto Ag@Ce NCs (*C*_0_ = 15 mg/L, *m* = 20 mg, *V* = 10 mL, pH = 6.8, AS = 200 rpm, *T* = 293 ± 1 K).

Contact time is a crucial parameter for the industrial application of adsorbents. An effective adsorbent must not only reach high adsorption capacities but also enable a fast process. The impact of contact time on the simultaneous adsorption of MB and SO dyes on Ag@Ce NCs is illustrated in Figure 10a,b. The results demonstrate that both %*RE* and *q_e_* peaked around 45 min and then unexpectedly decreased by 180 min. The initial rise within the first 45 min is due to the availability of active sites on the adsorbent at the start. Afterward, the surface became saturated. Generally, adsorption increases rapidly as dye molecules fill available sites, reaching equilibrium within a few tens of minutes. Salem et al. (2022) found that the magnetite/Ag nanocomposite reached equilibrium for the SO and MB binary mixture in approximately 40 min [60]. Similarly, Elsharif et al. (2021) observed SO adsorption on biomass leveling off at around 40 min [59], and Khalili et al. (2018) reported about 55 min as the optimal contact time for MB adsorption on a cellulose/MgO composite [65]. In each case, extending contact time did not significantly increase the adsorption. In some systems with slower kinetics, reaching equilibrium may take longer (e.g., 90–120 min for MB adsorption on polymeric nanoparticles) [66], but still shows a steady approach to equilibrium.

Conversely, in this binary system, the decrease in adsorption after 45 min indicates that equilibrium was reached and then partially reversed. This decline in removal suggests that some adsorbate molecules desorb or redistribute over time. Such behavior may result from the reversible nature of surface binding; once saturation occurs, dye molecules with weaker affinity may be released back into the solution or displaced. This pattern may be inherent to the properties of the adsorbent, as a similar trend was observed in the removal of CV dye using peanut husk–cellulose–Ag NCs, where the adsorption efficiency peaked at 90 min before declining [21]. Additionally, a recent study by Handayani et al. (2024) found that high adsorption of MB was achieved at 60 min, after which it decreased over time [67]. They explained this decline as the result of continued collision interactions between the biosorbent and the adsorbate, causing the dye to be released from the active sites of the adsorbent [67].

#### 3.2.4. Effect of Initial Concentration of Dyes

**Figure 11:**
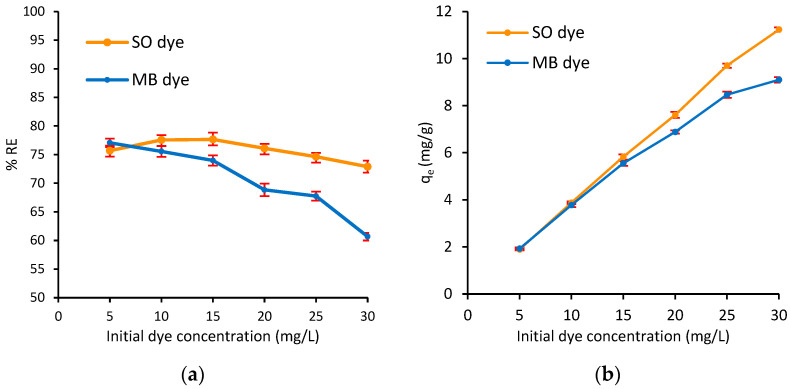
Effect of initial dye concentration on the (**a**) adsorption capacity and (**b**) simultaneous removal efficiency of MB and SO dyes onto Ag@Ce NCs (*m* = 20 mg, *V* = 10 mL, pH = 6.8, *t* = 45 min, AS = 200 rpm, *T* = 293 ± 1 K).

One of the most effective parameters in the adsorption process is the initial dye concentration. Therefore, the impact of varying the concentration of both MB and SO (5.0, 10, 15, 20, 25, 30 mg/L) on their adsorption onto Ag@Ce NCs was investigated. The experimental results are shown in Figure 11, indicating that as the initial concentration of MB increased from 5.0 to 30 mg/L, the %*RE* decreased from 77.1% to 60.7%. In contrast, the %*RE* of SO initially slightly increased from 75.7% to 77.7.% as the dye concentration increased from 5.0 to 15 mg/L, then slightly declined to 72.9% with further increase in SO concentration (Figure 11a). Meanwhile, the *q_e_* increased in both cases (e.g., MB and SO dyes) as the initial dye concentration increased from 1.93 to 9.10 mg/g for MB and from 1.89 to 11.2 mg/g for SO (Figure 11b). Generally, with increasing dye concentration, more dye molecules are present, while the fixed number of active sites becomes relatively saturated, leading to a decrease in removal efficiency. However, the higher concentration gradient drives more dye onto the adsorbent surface, thereby increasing the adsorption capacity [21,57,68]. Similar concentration effects have been reported in the literature. For example, various biomass adsorbents (cellulose, chitosan, fruit peels) typically show near-complete removal at low dye concentration and decreasing removal at higher concentrations due to site saturation. Additionally, our previous results from a study on similar peanut-husk cellulose-Ag for crystal violet (CV) showed that the removal of CV increased nearly linearly from 5.0 to 15 mg/L and then remained constant at 25 mg/L [21]. CV behavior reflects our SO behavior (initially increased, then slightly decreased). Similarly, citrus peel cellulose almost completely removed MB at 5.0 mg/L but demonstrated lower removal at 25 mg/L [69].

The difference in the adsorption behavior of MB and SO in the binary system with increasing initial concentrations can be explained by differences in their molecular size and structure (Table 1). The SO molecule, being larger, showed a slight increase up to 15 mg/L, followed by a minor decline, likely due to steric hindrance and early surface saturation. In contrast, the MB molecule exhibited a continual decrease in removal efficiency as the concentration increased, reflecting faster site saturation due to its smaller size and more efficient packing. However, for both dyes, the adsorption capacity (*q_e_*) increased significantly with concentration, consistent with enhanced mass transfer and accumulation on available sites. These trends align with the literature, which highlights the role of dye size in adsorption efficiency and site utilization [57,70,71].

#### 3.2.5. Effect of Ionic Strength

**Figure 12:**
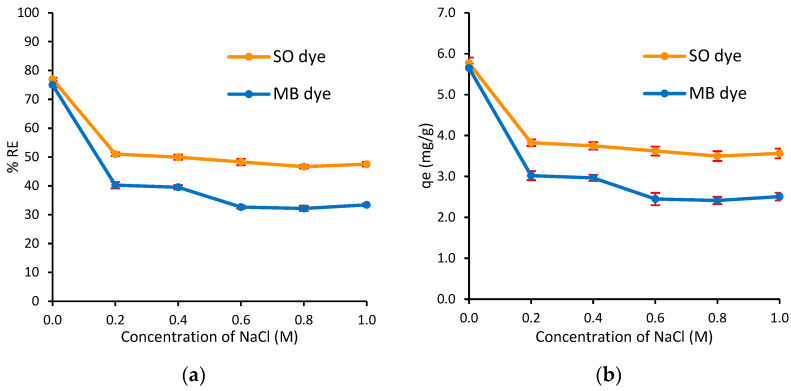
Effect of ionic strength on the (**a**) adsorption capacity and (**b**) simultaneous removal efficiency of MB and SO dyes onto Ag@Ce NCs (*C*_0_ = 15 mg/L, *m* = 20 mg, *V* = 10 mL, pH = 6.8, *t* = 45 min, AS = 200 rpm, *T* = 293 ± 1 K).

The presence of cations and anions in wastewater effluents significantly impacts treatment efficiency. This study evaluated the effect of ionic strength on the removal of MB and SO dyes onto Ag@Ce NCs at varying concentrations of NaCl solution from 0.2 to 1.0 M, and the results are shown in Figure 12. The results indicated that increasing NaCl concentration up to 1.0 M led to a decrease in the %*RE* from 75.3% to 33.4% for MB dye and from 76.9% to 47.5% for SO dye in the binary system (Figure 12a). A similar decrease was observed in adsorption capacity (*q_e_*) (Figure 12b). This reduction is attributed to the screening and competitive effects of added salt on the adsorbate–adsorbent interaction. Na^+^ ions compete with cationic dyes for negatively binding sites such as carboxylate groups on cellulose, while Cl^−^ ions may weakly interact with cationic dyes in solutions, thereby decreasing their availability for adsorption [72,73]. Comparable behavior has been reported for other nanomaterials; for example, K^+^ ions were found to significantly reduce MB adsorption on clay NPs due to site competition [72]. From an application standpoint, these findings emphasize that saline conditions are typically found in textile wastewater (0.02 and 0.17 M NaCl) [74] could decrease dye removal by 30–40%. Therefore, the design of treatment systems using Ag@Ce NCs should consider salt interference, potentially by pre-increasing the adsorbent dosage or integrating pre-treatment steps to mitigate the ionic effect.

#### 3.2.6. Effect of Temperature and Thermodynamic Parameters

**Figure 13:**
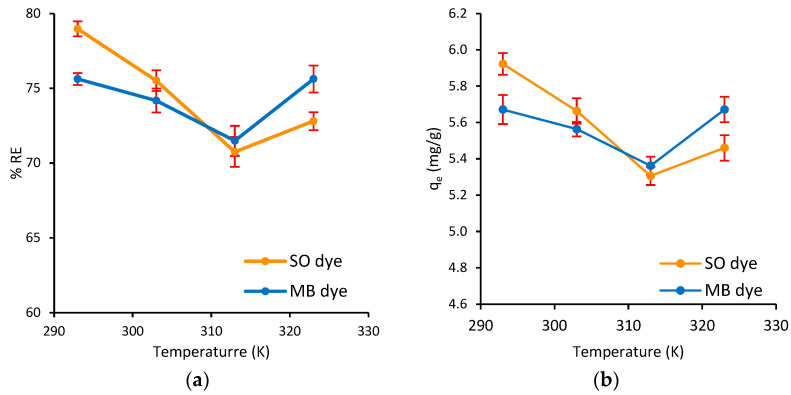
Effect of temperature on the (**a**) adsorption capacity and (**b**) simultaneous removal efficiency of MB and SO dyes onto Ag@Ce NCs (*C*_0_ = 15 mg/L, *m* = 20 mg, *V* = 10 mL, pH = 6.8, *t* = 45 min, AS = 200 rpm).

Temperature is a crucial physicochemical parameter because it influences the nature of the reaction. Additionally, it can significantly affect the adsorption rate, either increasing or decreasing it [75]. The effect of temperature on the simultaneous adsorption of MB and SO dyes onto Ag@Ce NCs was examined over a temperature range of 293–323 K to understand the thermodynamic adsorption behavior. As shown in Figure 13a,b, %*RE* and *q_e_* decreased as the temperature increased from 293 to 313 K, followed by a notable increase at 323 K. The initial decline suggests a predominance of exothermic processes, indicating that adsorption is greater at lower temperatures. The subsequent rise at 323 K could be attributed to a shift in the adsorption mechanism or the activation of additional favorable interaction sites at higher temperatures. This irregular trend may be interpreted as combination of predominantly physisorption at lower temperatures, where dye molecules bind weakly to Ag@Ce NCs via van der Waals forces or ion exchange, and chemisorption at higher temperatures, where stronger binding interactions and new active sites become accessible, thus improving removal efficiency and adsorption capacity [76]. The same adsorbent exhibited similar behavior in adsorbing CV dye when the temperature was increased from 298 K to 318 K, with a corresponding decrease in removal efficiency. However, adsorption was not measured at temperatures above 318 K in that study [21].

The thermodynamic results in Table 3 show that the adsorption of both MB and SO onto Ag@Ce NCs is spontaneous, as indicated by the negative Δ*G*° values at all temperatures. The decrease in the negativity of Δ*G*° with increasing temperature suggests that adsorption becomes less favorable at higher temperatures until 303 K, after which it becomes more favorable again at 323 K. This indicates a change in the thermodynamic behavior of both systems (e.g., MB and SO dyes). It implies that adsorption becomes more favorable at temperatures above 313 K, possibly due to increased molecular mobility or the activation of additional binding sites on the adsorbent surface. The Δ*G*° values ranging from 0 to −20 kJ/mol indicate that a physisorption mechanism mainly drives the adsorption process [77,78]. Thus, the adsorption of MB and SO on Ag@Ce NCs is a physical adsorption process. Moreover, the negative values of Δ*H*° presented in Table 3 for both dyes indicate an exothermic adsorption process, while slightly negative Δ*S*° values imply minimal structural change in the Ag@Ce NCs during dye adsorption. The low values of Δ*H*° for the adsorption of MB and SO dyes on Ag@Ce NCs are due to electrostatic attraction between the positively charged dye molecules and the negatively charged hydroxyl groups on Ag@Ce NCs, indicating physical adsorption rather than strong molecular interactions, and the specific properties of the adsorbent materials [20,79].

#### 3.2.7. Kinetic Studies

The adsorption kinetics illustrate how the process evolves over time, and the rate of adsorption significantly influences its efficiency and cost in industrial applications. In an adsorption experiment, contact time refers to the duration required for the maximum dye concentration to reach equilibrium with the surface of the adsorbent [60]. The adsorption kinetics of the binary system of MB/SO on Ag@Ce NCs were studied under stirring at 293 K. Linearized PFO and PSO models were applied, and the results are presented in Table 4. The PSO model provided a consistently better fit, with higher correlation coefficients (R^2^) and predicted *q_e_* values closer to the experimental data than the PFO. This superior fit suggests that the adsorption rate is primarily governed by surface-controlled interactions rather than by simple diffusion. Similar observations of PSO kinetics in binary dye systems have been reported in the literature [60,80,81].

#### 3.2.8. Isotherm Studies

Langmuir, Freundlich, and Temkin isotherm models were applied to investigate the adsorption of MB and SO from a binary solution on Ag@Ce NCs at 293 K. Table 5 presents the isotherm parameters related to this study. The Langmuir isotherm was the best fit for both dyes out of the three models, as indicated by the high correlation coefficients (R^2^ = 0.9976 for MB and 0.9969 for SO). This means that the adsorption process primarily follows a monolayer coverage pattern on a smooth surface with uniform active sites that have the same energy. In this model, each molecule that sticks to the surface interacts with it in a way that is independent and uniform, with no lateral interaction [60]. The highest monolayer adsorption capacities (*q_m_*) observed were 17.99 mg/g for MB and 14.90 mg/g for SO. This indicates that MB adsorbs slightly more under competitive conditions. However, Langmuir equilibrium constants (*K_L_*) were nearly the same for both dyes (0.1056 L/mg for MB and 0.1095 L/mg for SO), suggesting that the two dyes have similar binding affinities. Along with the comparable *q_m_* values, this suggests that Ag@Ce NCs do not preferentially select one dye over the other, allowing both to compete equally for surface sites. This finding is consistent with other research on cationic dyes adsorbed onto non-selective nanocomposite materials. Nonetheless, slight preferences for MB have been associated with differences in molecular structure, charge distribution, and the possibility of π-π stacking interactions with aromatic regions [82].

The Freundlich model also fits the experimental data well (R^2^ ≈ 0.97), but not as well as the Langmuir model, which suggests that the surface is not uniform (Table 5). The Freundlich constants 1/*n_F_* were 0.6788 for MB and 0.7004 for SO. Both values were between 0 and 1, indicating that the conditions for adsorption were favorable and that the surface was likely heterogeneous, allowing for multilayer adsorption [29]. This model assumes that the sites where things stick to the surface have different affinities and that the energy required to adhere to the surface decreases logarithmically as the surface becomes more covered. The slightly lower 1/*n_F_* value of MB indicates more heterogeneous binding sites for MB, potentially due to stronger dye-surface or dye-dye interactions. The Temkin model further supports these interpretations by providing an intermediate fit as presented in Table 5 (R^2^ = 0.9867 for MB and 0.9719 for SO). It assumes that the heat of adsorption decreases linearly with surface coverage because of interactions between the adsorbent and adsorbate. The Temkin binding constant (*b_T_*) was slightly higher for SO (0.7993) than for MB (0.7555), which suggests marginally stronger interactions for SO. Meanwhile, the heat of adsorption parameter (*B_T_*) was slightly greater for MB, indicating minor differences in energy profiles between the two dyes on the surface of Ag@Ce NCs [30].

#### 3.2.9. Adsorption Mechanism

**Figure 14:**
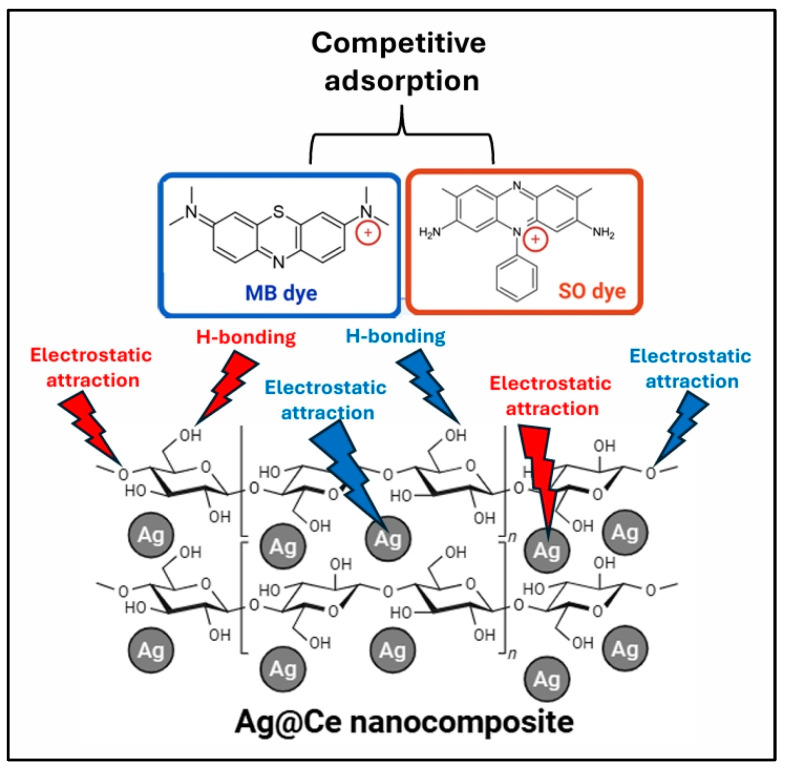
A schematic illustrating the adsorption of MB and SO molecules onto Ag@Ce NPs.

As observed from the adsorption results, MB and SO have similar adsorption capacities, which suggests they compete equally for the active sites on the adsorbent. In other words, the Ag@Ce NCs do not preferentially adsorb one dye over the other; they provide a non-selective surface where both dyes bind to a similar extent. If both dye molecules were present together, they would likely share the available adsorption sites almost equally, rather than one dye displacing or dominating the other. Studies on binary dye systems support this interpretation; for example, simultaneous adsorption of MB and SO on iron oxide nanoparticles resulted in similar maximum capacities for each dye (around 89–92 mg/g) [64]. These findings confirm that both dyes have comparable affinity for the adsorbent surface, leading to similar uptake when competing for binding sites. Essentially, the surface-active sites are equally accessible to MB and SO, so both dyes are adsorbed to a comparable degree under the same conditions. This lack of strong selectivity indicates similar competition, meaning each dye molecule is equally likely to occupy a given adsorption site [64]. The observed equal adsorption behavior can be explained by the similar mechanisms through which the dyes interact with the adsorbent. Both MB and SO are cationic (positively charged) aromatic dyes that interact with the adsorbent similarly. The cellulose-based adsorbent (especially when modified with silver nanoparticles) provides sites that are likely to carry negative charges or polar functional groups. Cationic dye molecules typically bind to negatively charged surface sites via electrostatic attractions or hydrophobic interactions [64]. MB, for instance, is known to adsorb through π–π interactions and electrostatic forces on cellulose composites [79]. SO, with a similar planar aromatic structure and positive charge, can attach using the same mechanisms, targeting the same types of active sites on the surface of the Ag@Ce NCs. Since both dyes depend on these common interactions, the material shows no strong preference for one over the other. Each dye molecule, whether MB or SO, finds the surface binding sites equally hospitable. Therefore, when the adsorbent dose increases (creating a surplus of binding sites relative to the amount of dye), both dyes saturate the available sites to a similar extent. Figure 14 summarizes the proposed adsorption mechanism, indicating that the adsorption capacities of MB and SO are similar, suggesting that the active sites on the adsorbent surface have comparable affinities for both dyes. This results in equal competition and similar uptake. It emphasizes that Ag@Ce NCs are non-selective toward these two organic cationic pollutants, as each dye molecule competes equally for adsorption.

#### 3.2.10. Desorption Study

**Figure 15:**
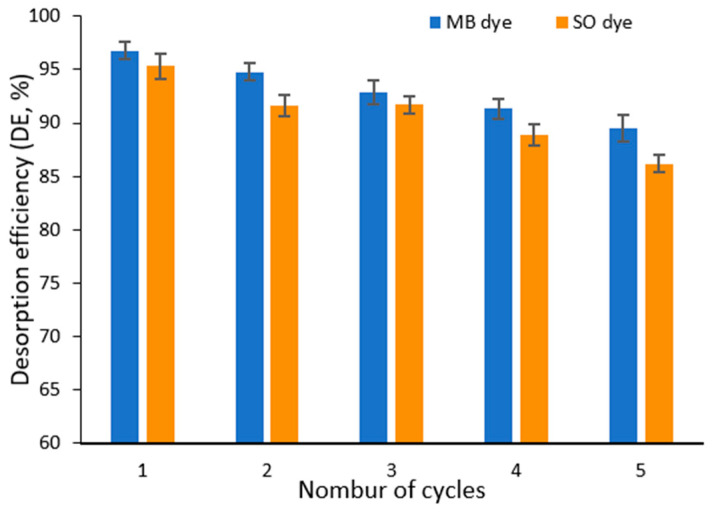
Desorption efficiency of MB and SO by Ag@Ce NCs in five adsorption–desorption cycles.

The regeneration ability and reusability of the adsorbent are crucial for its practical application in treating dye-contaminated wastewater. In this study, the regeneration performance of Ag@Ce NCs was evaluated through repeated adsorption–desorption cycles under acidic conditions using 0.1 M H_2_SO_4_. This condition was selected because it decreases the adsorption of MB and SO at low pH, facilitating dye removal. As shown in Figure 15, the adsorbent maintained a high desorption efficiency of about 89.0% for MB and 86.2% for SO after five cycles, which is nearly 76% and 71% of its original effectiveness for the removal of MB and SO, respectively (Figure S4). These findings demonstrate the good regeneration ability and stability of Ag@Ce NCs, highlighting their cost-effectiveness and environmental benefits for repeated use in water treatment. Importantly, the similar adsorbent synthesized in a previous study showed a regeneration efficiency of around 88.0%, further confirming the reusability of this type of nanocomposite material [21]. This finding indicates that the adsorbent is easily regenerable and possesses significant promise for wastewater treatment applications.

## 4. Conclusions

Ag@Ce NCs were synthesized using a simple, plant-assisted method and demonstrated excellent performance in simultaneously removing MB and SO dyes. Structural analyses confirmed successful Ag integration within the cellulose framework, effective delignification, and partial removal of hemicellulose. FTIR spectra after adsorption showed minimal changes, indicating the polymer backbone remained intact. TEM images displayed mainly spherical Ag nanoparticles (25–41 nm), evenly distributed and stabilized by cellulose, while EDX and XRD verified the expected composition and presence of metallic Ag. Electrokinetic studies (pH_pzc_ = 5.0; zeta potential = −11 mV) explain strong adsorption under neutral to alkaline conditions through electrostatic attraction to cationic dyes. Importantly, the adsorption process was rapid, reaching equilibrium within 45 min, was best described by the Langmuir model (indicating monolayer adsorption), and showed clear salt sensitivity, confirming the dominant role of electrostatic interactions. The process follows PSO kinetics, consistent with exothermic physical interactions involving electrostatic forces and hydrogen bonds. The nanocomposites retained about 75–70% of their initial capacity after five regeneration cycles, demonstrating durability and cost-effectiveness. Overall, Ag@Ce NCs are promising, eco-friendly adsorbents for dye-contaminated wastewater, with future research suggested to evaluate real effluents, salinity tolerance, and long-term operational stability.

## Figures and Tables

**Table 1:** The main characteristics of the dyes (adsorbates).

Dye	Methylene Blue, MB	Safranine O, SO
Type	Basic blue 9, C.I. 52,015, cationic	Basic red 2, C.I. 50,240, cationic
Phase	Solid	Solid
Molecular formula	C_16_H_18_ClN_3_S	C_20_H_19_ClN_4_
Molecular weight	319.85 g/mol	350.85 g/mol
Chemical Structure	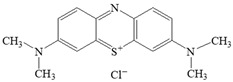	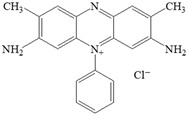
Color	Blue	Redish
λ_max_	664 nm	522 nm

**Table 2:** Mathematical equations of the isotherm models used in the study of MB and SO adsorption onto Ag@Ce NCs.

Isotherm Model	Equations		Parameters	Ref.
Langmuir	$\frac{1}{q_{e}}=\frac{1}{q_{max}}+\frac{1}{q_{max}\cdot K_{L}}\cdot \frac{1}{C_{e}}$	(6)	$q_{e}$ : equilibrium adsorption capacity (mg/g) $q_{max}$ : maximum monolayer adsorption capacity from Langmuir model (mg/g) $K_{L}$ : Langmuir constant (L/mg) $C_{e}$ : remaining concentration of the dyes in the solution (mg/L)	[28]
Freundlich	$lnq_{e}=lnK_{F}+\frac{1}{n_{F}}lnC_{e}$	(7)	$q_{e}$ : equilibrium adsorption capacity (mg/g) *K_F_*: Freundlich adsorption constants indicative of adsorption capacity (mg/g) $\frac{1}{n_{F}}$ : heterogeneity factor $C_{e}$ : remaining concentration of the dyes in the solution (mg/L)	[29]
Temkin	$q_{e}=B_{T}lnK_{T}+B_{T}lnC_{e}$	(8)	$K_{T}$ : Temkin isotherm equilibrium binding constant (L/g) $B_{T}$ : maximum binding heat of sorption (kJ/mol) *R*: ideal gas constant (0.008314 kJ/mol K) $b_{T}$ : binding energy *T*: absolute Temperature (K)	[30]
	$B_{T}=\frac{RT}{b_{T}}$	(9)		

**Table 3:** Thermodynamic Parameters of MB and SO adsorption onto Ag@Ce NCs.

Temperature (K)	MB Dye			SO Dye		
	Δ*G*° (kJ/mol)	Δ*H*° (kJ/mol)	Δ*S*° (J/mol K)	Δ*G*° (kJ/mol)	Δ*H*° (kJ/mol)	Δ*S*° (J/mol K)
293	−1.07	−1.21	−8.99	−1.53	−10.05	−29.46
303	−0.91			−1.091		
313	−0.59			−0.49		
323	−1.18			−0.78		

**Table 4:** Kinetic parameters for the adsorption of MB and SO in a binary solution on Ag@Ce NCs (2.0 g/L) at 293 K.

Dyes	PFO			PSO			*q_e_* (exp.) (mg/g)
	*q_e_* (mg/g)	*k*_1_ (min^−1^)	R^2^	*q_e_* (mg/g)	*k*_2_ (g/mg min^−1^)	R^2^	
MB	0.54	1.54 × 10^−^^2^	0.990	5.52	51.67 × 10^−2^	0.998	5.65
SO	0.47	1.90 × 10^−2^	0.946	5.70	23.99 × 10^−2^	0.999	5.77

**Table 5:** Adsorption isotherms parameters for the adsorption of MB and SO in single and binary solutions on Ag@Ce NCs (2.0 g/L) at 293 K.

Dye	Langmuir			Freundlich			Temkin			
	*q_m_*	*K_L_*	R^2^	*K_F_*	1/*n_F_*	R^2^	*K_T_*	*B_T_*	*b_T_*	R^2^
MB (binary)	17.98	10.56 × 10^−2^	0.998	1.95	0.68	0.971	13.67 × 10^−2^	3.22	0.76	0.987
SO (binary)	14.90	10.95 × 10^−2^	0.997	1.64	0.70	0.989	8.56 × 10^−2^	3.05	0.80	0.972
MB (single)	4.10	14.09 × 10^−2^	0.999	7.82	0.24	0.998	0.57 × 10^−2^	1.47	1.66	0.998
SO (single)	4.57	9.90 × 10^−2^	0.999	7.63	0.22	0.997	0.40 × 10^−2^	1.35	1.80	0.997

## Data Availability

The original contributions presented in this study are included in the article/Supplementary Material. Further inquiries can be directed to the corresponding authors.

## Supplementary Materials

The following supporting information can be downloaded at https://www.mdpi.com/article/10.3390/polym17182555/s1. Figure S1: UV-Vis absorption spectra of binary solution (MB/SO) before and after adsorption on Ag@Ce NCs using different dosages (*C*_0_ = 15 mg/L, *V* = 10 mL, pH = 6.8, *t* = 45 min, AS = 200 rpm, *T* = 293 K); Figure S2: UV-Vis absorption spectra of binary solution (MB/SO) before and after adsorption on Ag@Ce NCs after different contact times (*C*_0_ = 15 mg/L, *V* = 10 mL, *m* = 50 mg, pH = 6.8, AS = 200 rpm, *T* = 293 K); Figure S3: UV-Vis absorption spectra of binary solution (MB/SO) before and after adsorption on Ag@Ce NCs using different initial concentrations of the dyes (*m* = 20 mg, *V* = 10 mL, pH = 6.8, *t* = 45 min, AS = 200 rpm, *T* = 293 K); Figure S4: UV-Vis absorption spectra of binary solution (MB/SO) before and after adsorption on Ag@Ce NCs using different concentrations of NaCl (*C*_0_ = 15 mg/L, *V* = 10 mL, *m* = 20 mg, pH = 6.8, *t* = 45 min, AS = 200 rpm, *T* = 293 K); Figure S5: UV-Vis absorption spectra of binary solution (MB/SO) before and after adsorption on Ag@Ce NCs at different temperatures (*C*_0_ = 15 mg/L, *V* = 10 mL, *m* = 20 mg, pH = 6.8, *t* = 45 min, AS = 200 rpm); Figure S6: Removal efficiency of MB and SO dyes in binary solution onto Ag@Ce NCs after five cycles of adsorption–desorption process (*C*_0_ = 15 mg/L, *m* = 5.0 g/L, pH = 6.8, *t* = 24 h, AS = 200 rpm, *T* = 293 ± 1 K).
