# Supplementary material for: Eco-Friendly Synthesis of Silver–Cellulose Nanocomposite Adsorbent from Agricultural Residues for Binary Dye System Remediation
Source: Polymers (Basel). 2025 Sep 22;17(18):2555. doi: 10.3390/polym17182555 (PMC12473418; doi:10.3390/polym17182555)
Supplement: Supplementary file 1 [file polymers-17-02555-s001.zip › polymers-3850787-supplementary.pdf]

Eco-Friendly Synthesis of Silver-Cellulose Nanocomposite Adsorbent from Agricultural Residues for Binary Dye System Remediation

Doaa S. Al-Raimi, Reem M. Alghanmi, Ghalia S. Aljeddani, and Ragaa A. Hamouda

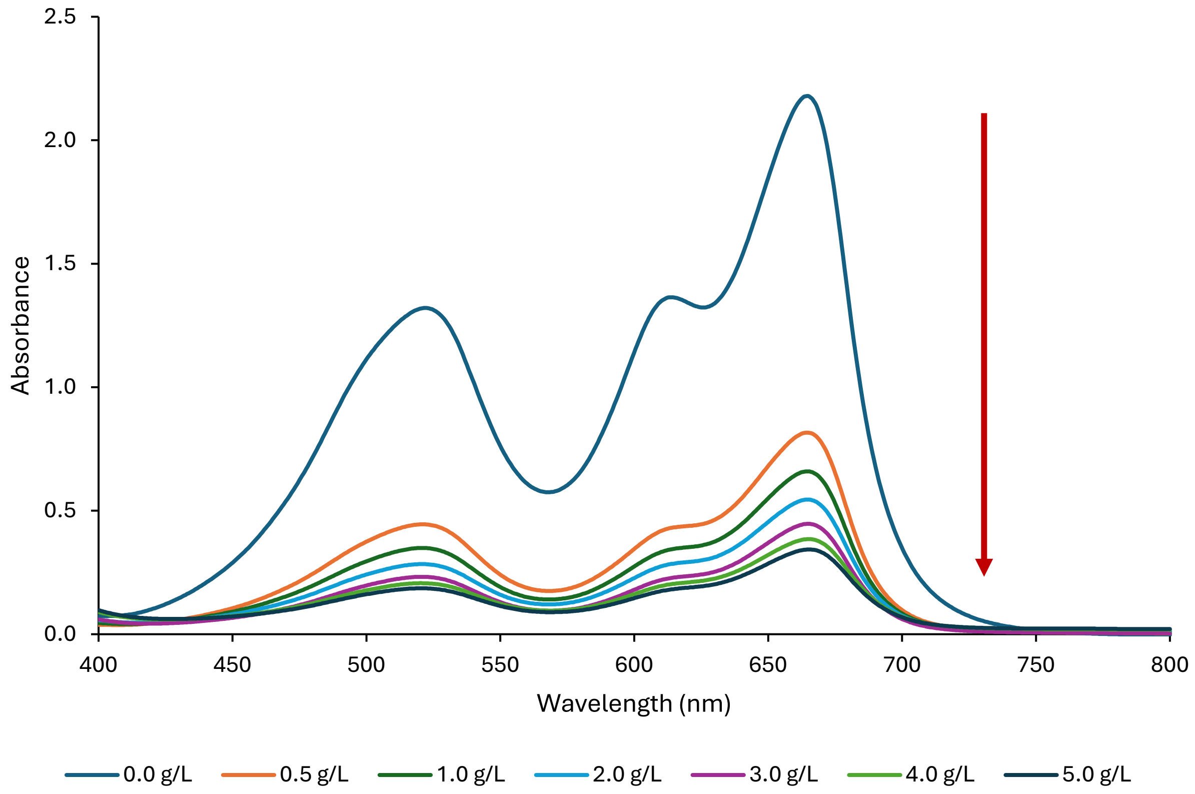

Figure S1. UV-Vis absorption spectra of binary solution (MB/SO) before and after adsorption on Ag@Ce NCs using different dosages (C_0 = 15 mg/L, V = 10 mL, pH = 6.8, t = 45 min, AS = 200 rpm, T = 293 K).

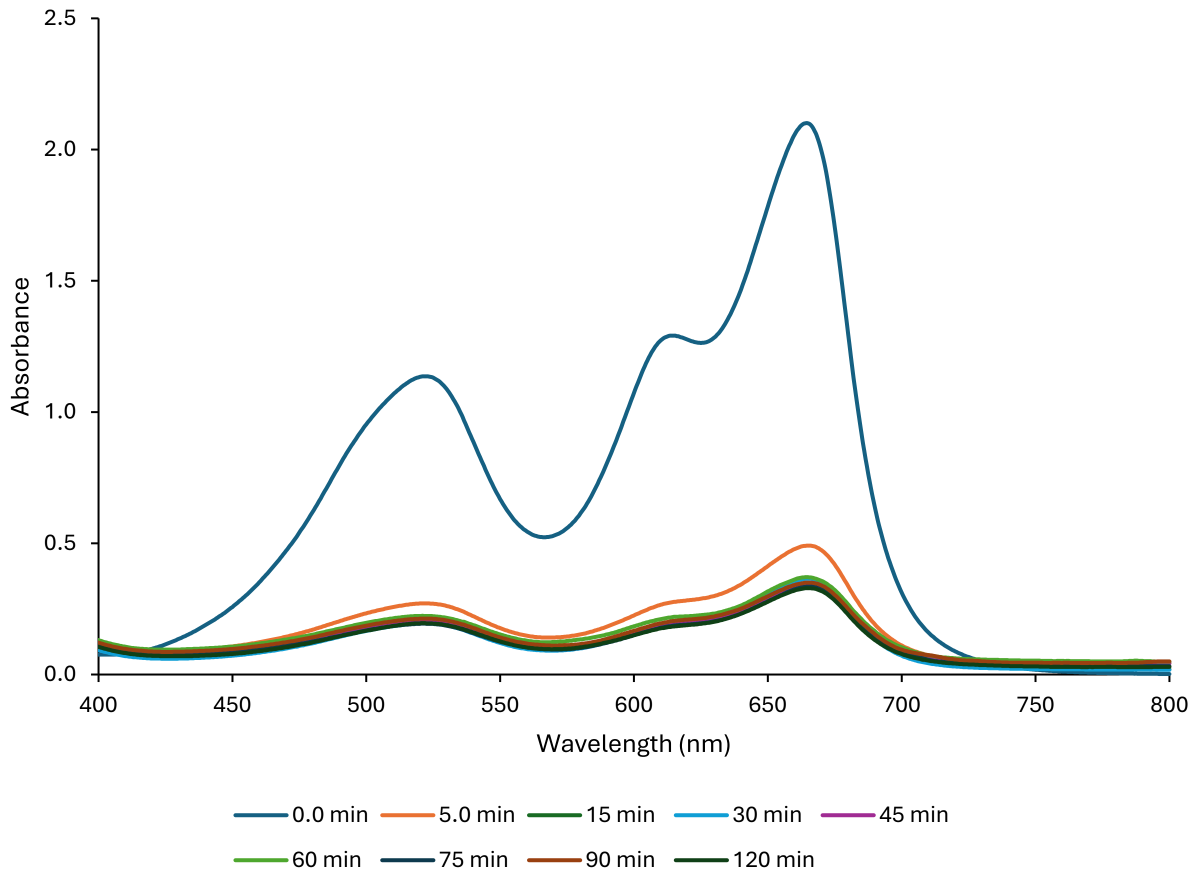

Figure S2. UV-Vis absorption spectra of binary solution (MB/SO) before and after adsorption on Ag@Ce NCs after different contact times (C_0 = 15 mg/L, V = 10 mL, m = 50 mg, pH = 6.8, AS = 200 rpm, T = 293 K).

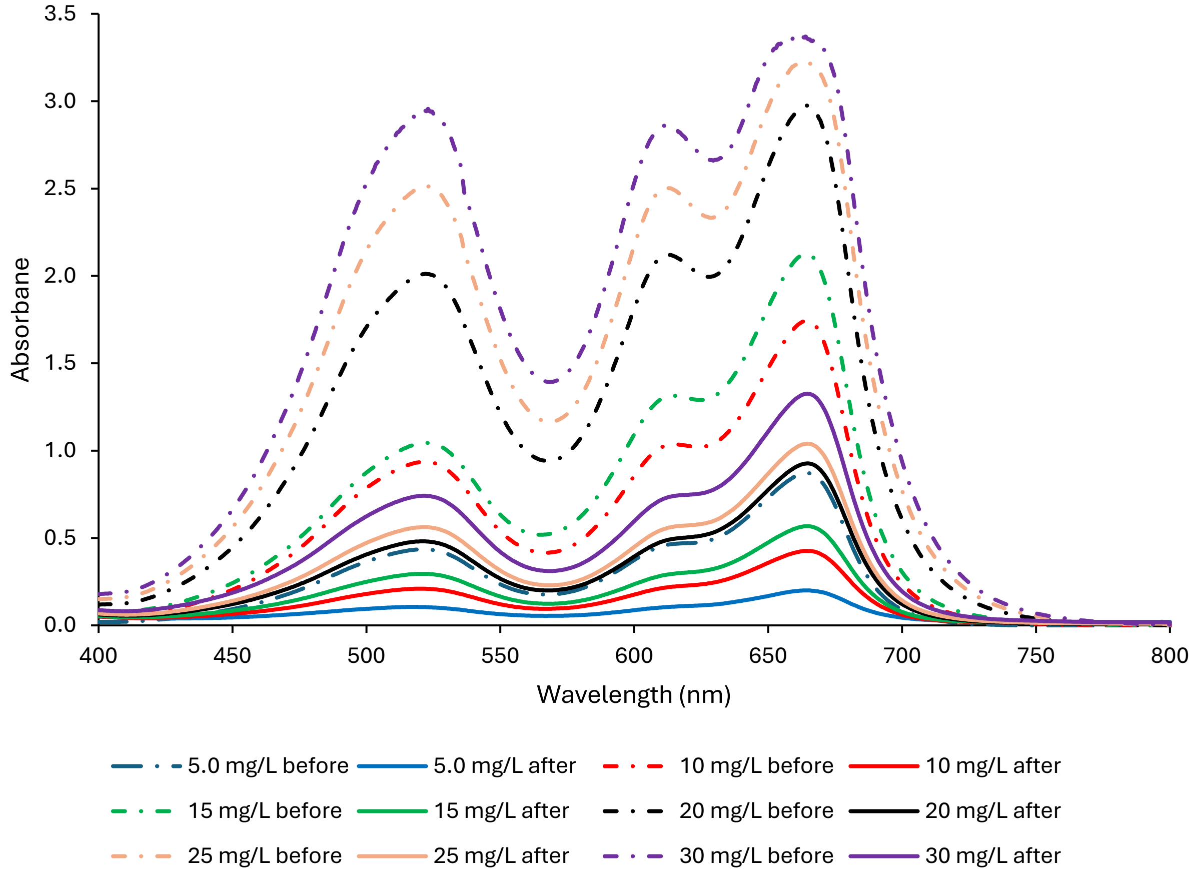

Figure S3. UV-Vis absorption spectra of binary solution (MB/SO) before and after adsorption on Ag@Ce NCs using different initial concentrations of the dyes (m = 20 mg, V = 10 mL, pH = 6.8, t = 45 min, AS = 200 rpm, T = 293 K).

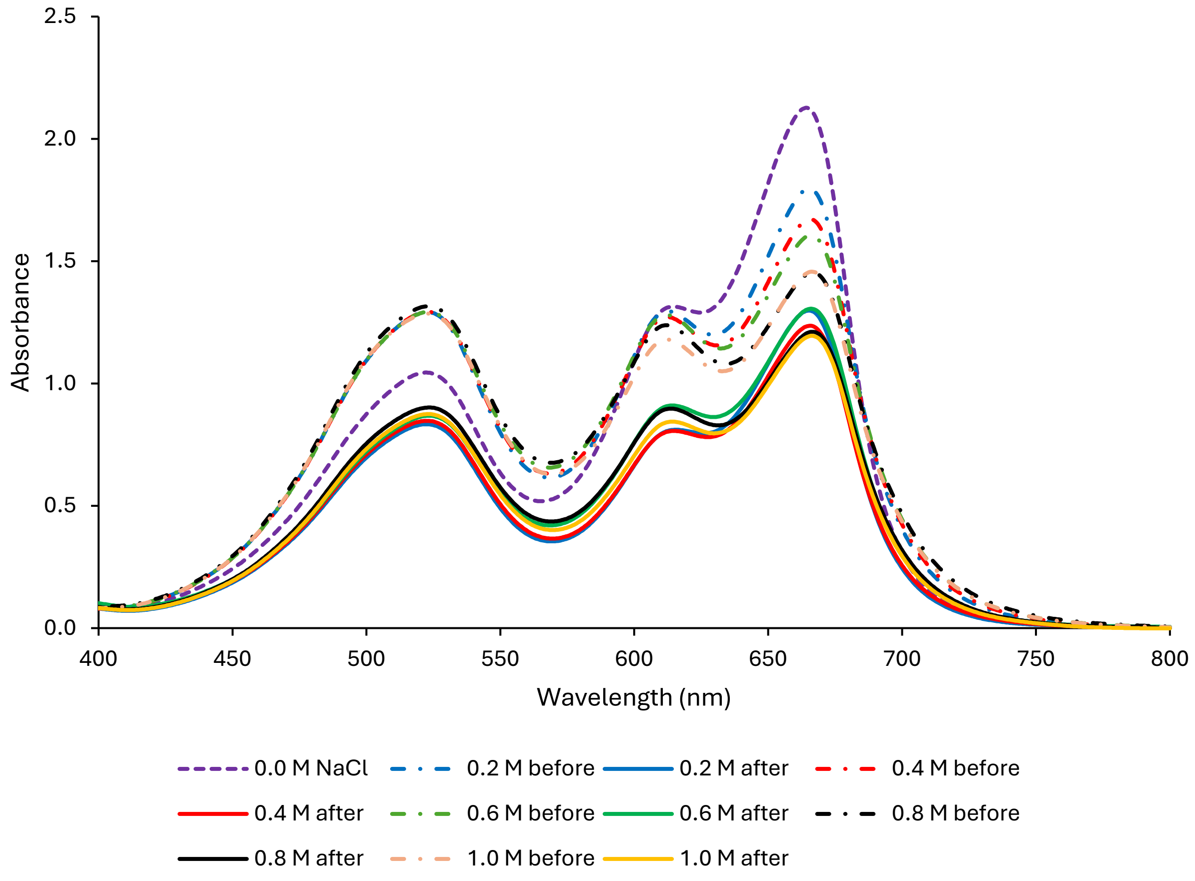

Figure S4. UV-Vis absorption spectra of binary solution (MB/SO) before and after adsorption on Ag@Ce NCs using different concentrations of NaCl (C_0 = 15 mg/L, V = 10 mL, m = 20 mg, pH = 6.8, t = 45 min, AS = 200 rpm, T = 293 K).

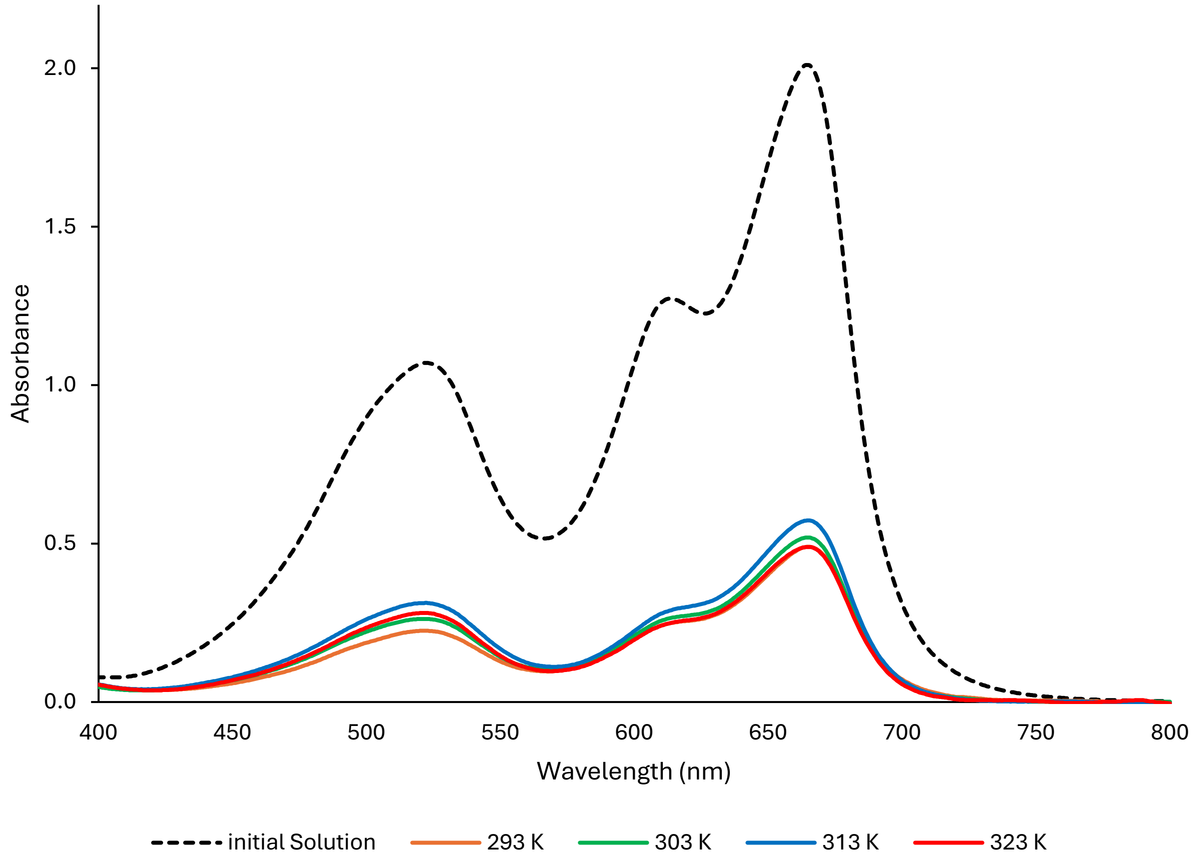

Figure S5. UV-Vis absorption spectra of binary solution (MB/SO) before and after adsorption on Ag@Ce NCs at different temperatures (C_0 = 15 mg/L, V = 10 mL, m = 20 mg, pH = 6.8, t = 45 min, AS = 200 rpm).

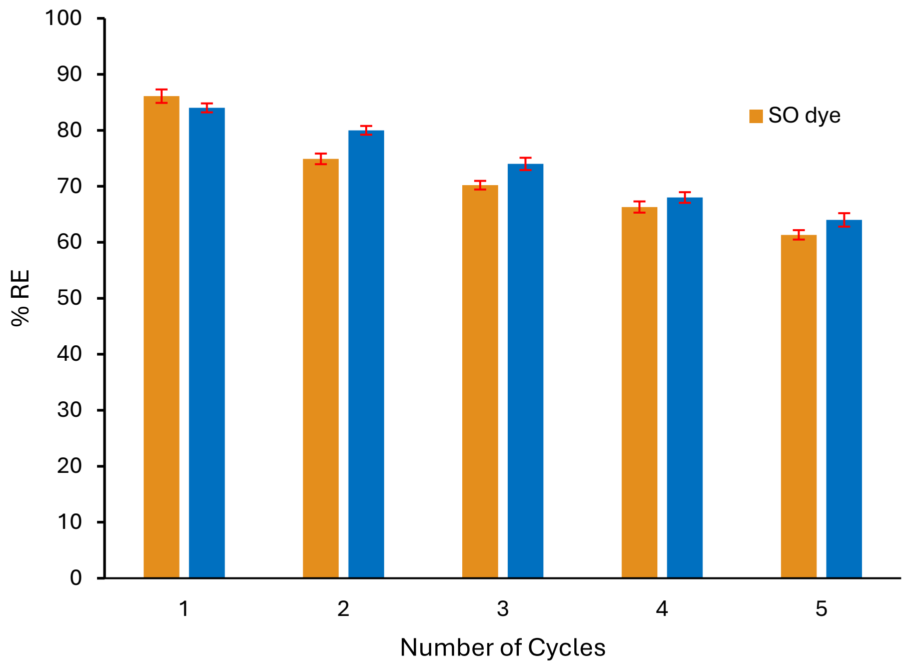

Figure S6. Removal efficiency of MB and SO dyes in binary solution onto Ag@Ce NCs after five cycles of adsorption-desorption process (C_0 = 15 mg/L, m = 5.0 g/L, pH = 6.8, t = 24 hours, AS = 200 rpm, T = 293±1 K).
